# Experiences with making diffraction image data available: what metadata do we need to archive?

**DOI:** 10.1107/S1399004713029817

**Published:** 2014-09-30

**Authors:** Loes M. J. Kroon-Batenburg, John R. Helliwell

**Affiliations:** a Crystal and Structural Chemistry, Bijvoet Center for Biomolecular Research, Utrecht University, Padualaan 8, 3584 CH Utrecht, The Netherlands; b School of Chemistry, Faculty of Engineering and Physical Sciences, University of Manchester, Brunswick Street, Manchester M14 9PL, England

**Keywords:** data exchange, data archiving, metadata, derived data, processed data, raw data

## Abstract

Recently, the IUCr (International Union of Crystallography) initiated the formation of a Diffraction Data Deposition Working Group with the aim of developing standards for the representation of raw diffraction data associated with the publication of structural papers. Archiving of raw data serves several goals: to improve the record of science, to verify the reproducibility and to allow detailed checks of scientific data, safeguarding against fraud and to allow reanalysis with future improved techniques. A means of studying this issue is to submit exemplar publications with associated raw data and metadata. In a recent study of the binding of cisplatin and carboplatin to histidine in lysozyme crystals under several conditions, the possible effects of the equipment and X-ray diffraction data-processing software on the occupancies and *B* factors of the bound Pt compounds were compared. Initially, 35.3 GB of data were transferred from Manchester to Utrecht to be processed with *EVAL*. A detailed description and discussion of the availability of metadata was published in a paper that was linked to a local raw data archive at Utrecht University and also mirrored at the TARDIS raw diffraction data archive in Australia. By making these raw diffraction data sets available with the article, it is possible for the diffraction community to make their own evaluation. This led to one of the authors of *XDS* (K. Diederichs) to re-integrate the data from crystals that supposedly solely contained bound carboplatin, resulting in the analysis of partially occupied chlorine anomalous electron densities near the Pt-binding sites and the use of several criteria to more carefully assess the diffraction resolution limit. General arguments for archiving raw data, the possibilities of doing so and the requirement of resources are discussed. The problems associated with a partially unknown experimental setup, which preferably should be available as metadata, is discussed. Current thoughts on data compression are summarized, which could be a solution especially for pixel-device data sets with fine slicing that may otherwise present an unmanageable amount of data.

## Abstract 2

A local raw ‘diffraction data images’ archive was made available and some data sets were retrieved and reprocessed, which led to analysis of the anomalous difference densities of two partially occupied Cl atoms in cisplatin as well as a re-evaluation of the resolution cutoff in these diffraction data. General questions on storing raw data are discussed. It is also demonstrated that often one needs unambiguous prior knowledge to read the (binary) detector format and the setup of goniometer geometries.

## 1. Introduction   

A Diffraction Data Deposition Working Group has been set up by the IUCr to consider the benefits, possibilities and costs of archiving raw diffraction images. Sensitivities to avoiding research malpractice are encouraging universities to establish their own data repositories for research and academic staff, providing ‘raw data archives’ that would complement the existing processed data archives. These archives would, however, be likely to have gaps in their global coverage arising from a lack of resources. Pioneering examples of raw data archives have been created in the USA, Australia and the UK. The JCSG (Joint Center for Structural Genomics) in the USA state on their website http://www.jcsg.org/datasets-info.shtml

The Joint Center for Structural Genomics has created a unique repository of X-ray crystallographic datasets for the structures that it has solved and deposited in the Protein Data Bank. This archive contains the experimental data and analyses from the data collection, data reduction, phasing, density modification, model building and refinement of JCSG structures. It also includes full sets of diffraction images for each of our deposited structures, enabling complete reconstruction of the data processing. In most cases, phasing was carried out either by SeMet, MAD or Molecular Replacement. These datasets are freely available to the scientific community for developing and testing new algorithms and benchmarking and teaching.

An early example of software improvement for processing of raw data (oscillation camera diffraction images recorded on film) was the comparative ‘round robin’ project of Helliwell *et al.* (1981 ▶). A federated repository for raw data, TARDIS, has been created in Australia (Androulakis *et al.*, 2008 ▶). In the UK, the Diamond Light Source, which has been operational for several years, is retaining all raw data (Ashton, 2011 ▶). In addition to these initiatives, we believe that a sufficiently large raw data archive, *i.e.* with reasonable global coverage, should be encouraged and would have major benefits.

In a number of countries, publishing data with a publication allows the researcher to comply with the grant conditions of funding agencies. Increasingly, funding agencies are requesting or requiring data-management policies (including provision for data retention and access) to be taken into account when awarding grants: see, for example, the Research Councils UK Common Principles on Data Policy (http://www.rcuk.ac.uk/research/Pages/DataPolicy.aspx) and the Digital Curation Centre overview of funding policies in the UK (http://www.dcc.ac.uk/resources/policy-and-legal/overview-funders-data-policies). It is worth noting, however, that these policies do not explicitly differentiate amongst derived, processed and raw data. We suggest that funding agencies might usefully develop a greater clarity of policy from one to the other stages of the ‘data pyramid’, with the raw data being the largest in file size and forming the base of the pyramid and with the derived data, being the smallest in file size and of most interest to the end user, at the pinnacle of the data pyramid; the processed data are mid-size files and are in the middle of the pyramid. The crystallography-community-derived, processed and raw data cases are described in §2.

A recent paper (Tanley, Scheurs *et al.*, 2013 ▶) that describes a joint effort of the Manchester and Utrecht groups to transfer and processes 11 data sets of cisplatin-bound or carboplatin-bound lysozyme crystals was linked *via* the journal’s webpage to a local raw data archive at Utrecht University (http://rawdata.chem.uu.nl) and was subsequently, basically immediately, mirrored at the TARDIS raw diffraction data archive in Australia, where the publication was seen. A digital object identifier for each data set underpinning a published paper at an archive local to where the data were measured is a plausible (*i.e.* most convenient) and economic model to move these data-archiving developments forward. The cost requirements of long-term storage and curation of digital data and large-bandwidth access for remote archives are important issues, but each solution requires provisions for a sufficient level of metadata detail to allow future use of the data. This paper addresses experiences with the transfer, archival and use of raw diffraction data.

In protein crystallography, X-ray diffraction data are often obtained from synchrotron beamlines that provide high-brilliance beams and rapid data collections, producing gigabytes of data within one beamline shift. Currently, home microfocus X-ray sources with matching multilayer optics and high-performance detectors are available and can compete with second-generation synchrotron beamlines, producing good-quality data sets but at a much lower byte rate. Future raw data-archiving policies should address both sources of data, but they may ultimately be combined somehow, for example by ‘cloud storage’. The end result of a diffraction experiment is a series of recorded diffraction data images. Metadata are contained in the header of these image files or internally on a server computer. The internal software provided by the detector manufacturer usually takes care of the necessary technical aspects, *i.e.* measuring apparatus and corrections such as those for detector non-uniformities, pixel distortions and parallax effects. Processing such image data remote from the equipment requires finding all of the necessary metadata.

The raw data archive in Utrecht led one of the maintainers of *XDS* (K. Diederichs) to re-integrate the data from crystals that we believed to simply contain fully bound carboplatin. This resulted in analysis of the anomalous electron densities near the Pt-binding sites, which whilst a signal-to-noise challenge were readily interpreted as partially occupied chlorines in cisplatin, *i.e.* a conversion product from carboplatin after high NaCl (10%) conditions. The collaboration that ensued also led to the scrutiny of and the use of several criteria to more carefully assess the diffraction resolution limit. Finally, in addition, the problems associated with a partially unknown experimental setup, which preferably should of course be available as metadata, are discussed.

## 2. Why store raw data?   

We offer a collection of reasons for depositing data and making them available alongside a scientific publication. At this stage, we make no distinction between derived, processed and raw diffraction data; we will make this distinction shortly. The global crystal structure databases have from their inception received coordinate data, *i.e.* derived data. They expanded in response to the community-expressed wish to accompany coordinate data with processed diffraction data, *i.e.* structure factors. The list below is not necessarily a complete list, but it provides a useful set of criteria and principles that are relevant, to a greater or lesser degree, across most probably all scientific fields.

(i) To enhance the reproducibility of a scientific experiment.
(ii) To verify or support the validity of deductions from an experiment.
(iii) To safeguard against error.
(iv) To better safeguard against fraud than is apparently the case at present.
(v) To allow other scholars to conduct further research based on experiments already conducted.
(vi) To allow reanalysis at a later date, especially to extract ‘new’ science as new techniques are developed.
(vii) To provide example materials for teaching and learning.
(viii) To provide long-term preservation of experimental results and future access to them.
(ix) To permit systematic collection for comparative studies.

In some cases these goals are adequately met by processed data. In other cases, whilst they may be satisfied by processed data, better results can be achieved using raw data. In a few cases, the raw data are essential (for example, in extracting new science from experiments that are not repeatable).

We now come to several linked, central, questions. When might the derived and/or processed diffraction data that are currently deposited in centralized databases become inadequate (*i.e.* when might the raw data become valuable)? How often might this be the case? What are the costs and benefits of retaining and having access to raw diffraction data? Where might raw diffraction data be most easily and cheaply housed?

A first, and most important, question is what is the diffraction resolution limit of any biological crystallography study? Were the processed diffraction data in effect artificially truncated at an arbitrary resolution limit even though the diffraction raw images extended to higher scattering angles, as they very often do? In particular then, the reason to preserve raw diffraction images is that it is not so easy to set a community-agreed standard for the edge of the diffraction pattern (the ‘diffraction resolution’). Blow (2002 ▶) refers to it very evocatively as ‘…the vexed question of deciding the practical limit of resolution’. The diffraction pattern fades basically owing to the atomic mobilities along with possible static disorder (called atomic displacement parameter effects), and in the case of X-rays and electrons as probes the finite size of the electron-charge cloud causes a further drop in the scattering of each atom. With neutrons the scattering, being from the much smaller nucleus, does not add to the atomic displacement parameter effect. In special cases, which are not uncommon, the diffraction resolution may be anisotropic owing to the nature of the overall quality of a crystal. In practice, one often uses a parameter descriptor that simply describes where the average diffraction spot intensities divided by their standard deviations σ [*i.e.* 〈*I*/σ(*I*)〉] decreases below 2.0. The community is keen, however, not to artificially cut the data here, as this would falsely eliminate diffraction spots even further from the centre of the diffraction pattern. Indeed, it is hoped that protein model-refinement programs should cope formally with the diffraction-pattern fade-out. This is not general practice, nor is it even championed by software writers, whether or not it is coded for in their mathematical algorithms. Indeed, as one watches the diffraction patterns as they are measured, occasional spot intensities do occur well beyond the obvious pattern edge. These occasional spots are known about but are deemed rare and so small in number to be considered inconsequential; but they are surely (or should surely be) of interest and potential help to better define our molecular models. The deletion of raw diffraction data and/or their loss owing to inadequate archiving means a loss to future possible revisions of molecular models using diffraction data beyond the actual analytic diffraction resolution of a given publication.

There are several other situations of strong interest for the preservation of raw diffraction images. Firstly, the processed diffraction data (structure factors) describe the diffraction structure amplitudes associated with the discrete spots. So, what do we perhaps ignore between the spots? It is apparent that significant amounts of scattered X-radiation may be measured between these Bragg-diffracted beams, the so-called diffuse scattering, yet this information is routinely discarded in crystal structure analysis.

Secondly, the processing of the Bragg diffraction spots themselves leads to an early decision by the crystallographer on just what the symmetry layout of a crystal actually is, namely its space-group symmetry, and which may not be appropriate.

Thirdly, there are situations of challenging cases in which the sample is actually a composite of two or more crystals and more than one diffraction pattern is then obviously visible in the raw diffraction image. The crystallographer will, by likely current practice, choose one such ‘crystal lattice’, typically the predominant one, and not the other(s); preserving the processed diffraction data of just that one crystal lattice obviously does not include the others, which are lost completely upon deletion of the raw diffraction data.

Fourthly, sometimes the raw diffraction data do not lead to any final interpretation in the hands of one crystallographer or laboratory. Such data could be made available, if a researcher finally chooses, to the wider community to attempt structure determination.

There is a further reason that is proposed for the utility of preserving raw diffraction data, namely the prevention of scientific fraud. The raw data would present a much greater hurdle against fabrication. The crystallographic community is somewhat divided on the effectiveness of this, however, in that it may ultimately prove achievable to fabricate raw diffraction data too.

## 3. Transferring and storing raw diffraction images   

**Figure 1:**
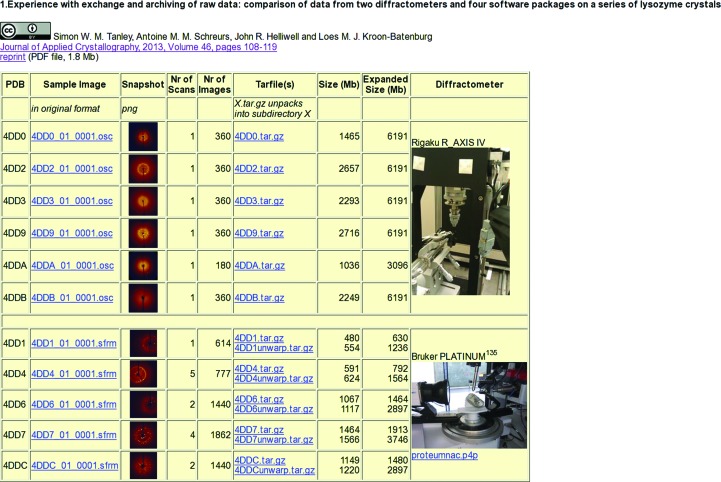
The web page at http://rawdata.chem.uu.nl from which raw data can be downloaded as gzip-compressed tar files. A sample image is shown for each data set and also a single image file is available in the original file format. For Bruker data both the original and unwarped data can be obtained. In the former case the p4p file is needed to unwarp images. Photos of the diffractometers can be useful in unravelling their setup.

In a previous paper (Tanley, Schreurs *et al.*, 2013 ▶), we published a detailed analysis of the diffraction data from 11 lysozyme crystals with bound platinum anti-cancer drugs collected in Manchester on a Rigaku R-AXIS IV image plate or on a Bruker CCD PLATINUM^135^ detector. These were processed by using the equipment’s software [either d*TREK (Pflugrath, 1999 ▶) or* PROTEUM*2 (Bruker)], *MOSFLM* (Leslie, 1999 ▶) using *SCALA* (Evans, 2006 ▶) for scaling, and *EVAL* (Schreurs *et al.*, 2010 ▶) using *SADABS* (Sheldrick, 1996 ▶) for scaling. The R-AXIS images (3000 × 3000 pixels) each take 18 MB of disk space, while the PLATINUM^135^ images (1024 × 1024 pixels in binned mode) take 1 MB. Owing to the high-redundancy data collection, a total of 35.3 GB of data were generated and were transferred to Utrecht, a process that took a few days. When the images were received in Utrecht after network transfer from Manchester they were immediately compressed using *ncompress*, reducing the R-AXIS image size to 3.8 MB and the PLATINUM^135^ image size to 800 KB. *ncompress* is public-domain software that uses the LZW (Lempel–Ziv–Welch) algorithm for lossless data compression (Welch, 1984 ▶). In future it may be advisable to use on-the-fly compression (*e.g.* scp -C in linux) during file transfer as well as a simple concatenation of the various data sets. Raw data linked to the paper can be found at http://rawdata.chem.uu.nl (Fig. 1 ▶). Two other papers (Tanley *et al.*, 2012 ▶; Tanley, Diederichs *et al.*, 2013*a* ▶) are based on additional data sets that will also be archived on the local repository in Utrecht.

For a research project that might take months, the time needed for file transfer is not an important issue. With the availability of inexpensive mass-storage devices, such amounts of data do not present a major obstacle either. However, a typical trip to a synchrotron beamline equipped with a PILATUS 6M detector and operating in fine-sliced mode yields tens of gigabytes of data that might need or be preferred to be transferred, stored and archived at the home laboratory; rarely are these data processed to a stage where it is sufficient to take only reduced (*h*, *k*, *l*, *I*) files, although this situation itself is changing. Currently the Australian TARDIS Store.Synchrotron project (https://www.rdsi.edu.au/rdsi-story-storesynchrotron) and the ISIS neutron source (https://data.isis.stfc.ac.uk/doi/INVESTIGATION/24079627/) are exemplars for data archiving. Furthermore Diamond Light Source has kept all raw data ever measured there on tape archives. But in general synchrotron and neutron facilities do not provide a permanent collection of all data measured there.

### 3.1. Costs of the storage of terabytes of data   

Archive storage of crystallographic data does not need fast access. Long-term storage is an important issue, which we might define as over five years, since funding agencies at present define data-management policy periods for data retention of five years, but without actively curating the storage system, be it USB drives, RAID systems or tapes, data will eventually become lost.

Hard drives may be acquired at less than 80 USD per terabyte. Simple RAID systems are relatively expensive and are not suitable for the storage of petabytes owing to the statistical likelihood of multiple drive failures (http://www.storagenewsletter.com/news/marketreport/why-raid-dead-for-big-storage-cleversafe).

Storage pods consisting of 4 TB hard drives with 180 TB in total can be obtained for as little as 2000 USD (https://blog.backblaze.com/180tb-of-good-vibrations-storage-pod-3.0/).

On the CCP4 Bulletin Board (ccp4bb) discussion thread on this in 2012 estimates were given that synchrotron installations wordwide produce 1600 TB of data each year (Holton, 2012 ▶). Storing these would cost somewhere between 18 000 and 160 000 USD per year for storage pods and USB drives, respectively, without active maintenance. Westbrook (2012 ▶) estimated that storage on a cheap RAID system would cost 960 000 USD for 1600 TB, which is more expensive than USB drives. Storing only the raw data associated with publications and PDB depositions was estimated to require only some 32 TB per year, and would cost roughly 350 USD on storage pods, 3200 USD on USB drives or 19 200 USD on a cheap RAID system. The non-RAID systems may require backup provisions and thereby at least double their costs.

One option would be to use commercial data storage ‘in the cloud’. Google offers this for 800 USD per 16 TB per month. The 1600 TB of data would then cost over 960 000 USD per year. To store the publication-associated data would cost ∼18 000 USD per year. In view of these estimates, a centralized archive, be it on RAID or cloud systems, may not be a realistic option, whereas local or federated repositories could choose the cheaper storage devices that can be kept alive with limited effort when locally maintained.

As mentioned in §[Sec sec3]3, data can be compressed by a ratio that depends on the byte-storage algorithm being used for writing the image files (see §[Sec sec3.2]3.2) and is absolutely necessary if we were to archive raw diffraction data. We also would have to consider the possibility of lossy data compression.

Holton (see http://bl831.als.lbl.gov/~jamesh/lossy_compression) applied a lossy-compression algorithm to ADSC images from a lysozyme crystal by splitting these into images that contain reflections and those that contain the background. The background images are then compressed in such a way that the noise level stays roughly the same and finally the two types of images are recombined. He achieved a compression ratio of 34 without changing the visual perception of the images and without changing the derived structure factors significantly [〈Δ*F*/σ(*F*)〉 = 0.6].

Ferrer *et al.* (1998 ▶) examined lossy data compression of CCD images with discrete cosine or wavelet transforms and also found little effect on integrated intensities except for weak reflections, which is clearly related to altered statistical noise in the background. The obvious advantages for network transfer and disk space could be outweighed by the (unforeseen) future need of raw images. One aspect could be the diffuse scattering of less ordered crystals (such as a normal protein crystal!) that occurs in areas that we normally designate background and which is weak and varies slowly. Currently, no one has established whether such compression algorithms will significantly affect diffuse intensities in recip­rocal space.

### 3.2. Metadata   

The metadata of the raw diffraction images stored at our local archive in Utrecht, highlighted in Fig. 1 ▶, are contained in the image header. However, not all image formats are equally informative. For example, we found that the Rigaku diffraction images did not provide all of the needed metadata information. A field in the header for the orientation of the spindle axis, for example, is reserved but did not contain a value. Assuming the spindle is perpendicular to the X-ray beam, it is obvious what the sense of rotation is, *i.e.* clockwise or anticlockwise, by looking at a few consecutive frames. Most data-processing software developers can find a consistent interpretation of the data, but preferably more metadata should be made available. The Rigaku Company has developed a new ASCII header type that contains all of the definitions for the orientations of the goniometer axes and for the detector axes in the laboratory frame, so that a comprehensive set of metadata is then provided.

The Bruker image format contains much more metadata, in particular the model of the goniometer and the goniometer rotation angles defined as Euler angles. However, we had to learn from previous data that the rotation directions for 2θ, ω and χ are opposite to that of ϕ.

Ideally, metadata should comprise the following: identification of the image format, number of pixels, pixel sizes, byte-storage architecture, baseline offset and handling of overflows, information on the corrections that are applied (dark current, distortion correction, non-uniformity correction), detector gain, goniometer axes orientations and rotation directions, and information on the experiment such as exposure time, number of repeats, oscillation axis and range, wavelength used, beam polarization, detector position (or beam position) and offsets. Another parameter that would be useful as part of the metadata is discussed by Owen *et al.* (2009 ▶). Knowing the incident beam flux allows the dose absorbed by the crystal to be estimated, and we recommend that the incident beam flux be given as metadata. The byte-storage architecture has to be obtained from the detector manufacturer. The authors of integration software such as d*TREK (Pflugrath, 1999 ▶), *DENZO*/*HKL*-2000 (Otwinowski & Minor, 1997 ▶), *MOSFLM* (Leslie, 1999 ▶), *XDS* (Kabsch, 2010 ▶) and *EVAL* (Schreurs *et al.*, 2010 ▶) have performed the same tedious unravelling of detector formats. To avoid having to undertake such efforts, the CBF/imgCIF format was developed (Bernstein, 2005 ▶; Bernstein & Hammersley, 2005 ▶). It provides a metadata structure in which all of the metadata can be found in one place. It consists of an ASCII imgCIF header and binary (CBF) or ASCII-based encoded data blocks. The binary format is reasonably (see later) space-efficient owing to the use of compression algorithms such as byte_offset compression, and it is useful for large images and for data transfer between collaborating groups. Three categories of data exist, ARRAY data, AXIS data and DIFFRN data, allowing a unique definition of how to interpret the data, and no prior knowledge would be required if all data items were filled in. This is often not the case, however; for example, PILATUS detector image files contain all relevant metadata in just a small comment line block, the so-called miniCBF format.

Bruker has made a plug-in in their *APEX* and *PROTEUM* software that can convert to proper imgCIF header and binary CBF format, completely adhering to the official specifications. The size of *APEX* images in native Bruker format and CBF format are roughly the same; they are seemingly both very efficient as they cannot be further compressed. We found that the PILATUS CBF files can be compressed to about one third of their initial size, normally being recorded in fine-slicing mode and having very little background noise, showing that the binary CBF format, using byte_offset compression, is not overly efficient. Several other compression algorithms are available in the CBF format that are probably more efficient but are rarely used. The updated version of byte_offset, nibble_offset, achieves lossless compressions of 12:1 with realistic MX data and has been made available for use with both CBF and NeXus (Bernstein, 2013 ▶). At the same time this means that PILATUS images recorded in fine-slicing mode can be compressed hugely, especially when using lossy compression algorithms such as those described above, without significant loss in the data, and that the concerns of archiving PILATUS data may not be fully justified. However, future data-storage challenges are on the way. The newly developed Eiger detector requires 72 MB of disk space for an uncompressed image and the current free-electron laser (FEL) at LCLS generates data at a rate of 20 TB per day, while with the European XFEL this is expected to increase by a factor of as much as of 100. However, in the case of the LCLS data many of the images are discarded for not containing any diffraction signal and thus the actual data sets to be processed may be much smaller in size. The metadata needed to describe them would be the same whether the data sets were large or small and so clarity at the outset with the metadata would benefit all raw data sets.

The next section describes the challenges that we faced in extracting metadata from image headers.

## 4. Data processing   

In response to our data-comparison paper and the setup of our local raw-data archive, K. Diederichs (University of Konstanz) reprocessed some X-ray diffraction data sets from lysozyme crystals containing carboplatin. These were the data sets with PDB codes 4dd9 (Rigaku R-AXIS images) and 4dd7 (Bruker PLATINUM^135^ images with kappa goniometer) from Tanley, Schreurs *et al.* (2013 ▶) and data set 4g4c from Tanley *et al.* (2012 ▶). Data set 4g4c is from an APEX2 detector (nonbinned), 60 pixels cm^−1^, 1024 × 1024 pixels with a fixed-χ goniometer. The raw data in our local archive were unwarped (corrected for distortion) using Bruker’s *FrameUtility* program and rewritten in a two-byte format because *MOSFLM* and *XDS* cannot read the Bruker native (u8) format. In addition, we changed the header with a Python script because we found that the header written by *Frame­Utility* contains more bytes than *MOSFLM* expects. This was caused by an error in *MOSFLM* in calculating the number of 512-byte blocks that have to be read in (this has been reported to the authors of *MOSFLM*) and by *FrameUtility* writing the end-of-header mark (crtl-Z–crtl-D) at the end instead of at the beginning of a line. As an example of how difficult the interpretation of incomplete and inaccurate metadata can be when trying to read raw images, we compile the discussion that K. Diederichs (KD), L. Kroon-Batenburg (LKB) and A. Schreurs (AS) had with respect to the 4g4c data in the Supporting Information^1^.

As a follow-up to the research performed with the archived data, additional X-ray diffraction data sets were collected in Manchester from hen egg-white lysozyme (HEWL) crystals co-crystallized with carboplatin without sodium chloride (Tanley, Diederichs *et al.*, 2013*b* ▶) to eliminate the partial conversion of carboplatin to cisplatin observed previously and were processed with *SAINT*, *EVAL* and *XDS*. The X-ray diffraction data resolution to be used for the model refinement was reviewed because the three processing programs may indicate different cutoff limits. The CC_1/2_ criterion implemented in *XDS* led to data being considered significant to 2.0 Å resolution, compared with the data only being able to be processed to 3.0 Å resolution using the Bruker software package (*SAINT*). Using paired protein model refinements and Cruickshank–Blow diffraction precision index (DPI) values based on the *R*_free_ value (Cruickshank, 1999 ▶; Blow, 2002 ▶), the resolution limit was fine-tuned to 2.3 Å. Interestingly, this was compared with results from the *EVAL* software package, which gave a resolution limit of 2.2 Å solely using 〈*I*/σ(*I*)〉 crossing 2 but of 2.8 Å based on the *R*_merge_ values (60%).

## 5. Detector gain and standard deviations   

Every detector converts the X-ray photons into an electronic signal that is read out and stored in an image file. The DQE (detector quantum efficiency) is a measure of the efficiency with which photons are detected and of the noise performance of the detector. It is defined as the signal-to-noise ratio of the output divided by that of the input. For an ideal detector this ratio would be 1.0. In practice, a variety of factors reduce this number. Waterman & Evans (2010 ▶) analysed and estimated all of the contributions to standard deviations of measured diffraction intensities recorded on CCD area detectors. A cascade of conversions, amplifications, transmissions, electronic noise and read-out noise adds to the standard deviations. Ideally, pixel intensities should be divided by the gain to obtain the X-ray photon counts, so that their standard deviations can be estimated using Poisson statistics. However, Waterman & Evans (2010 ▶) clearly showed that such Poisson standard deviations are heavily underestimated.

Similarly, Leslie (1999 ▶) and Popov & Bourenkov (2003 ▶) showed that the variance of integrated intensities behaves other than according to Poisson distributions and can be described by a second-order polynomial function in *I*: σ^2^ = *k*_0_ + *k*_1_ *I* + *k*_2_ *I* ^2^. The second term represents the error estimate from Poisson statistics (σ = *I* ^1/2^) corrected for the gain and Lorentz polarization, with the other two terms accounting for Poisson error contributions of the background scattering as well as read-out noise and so-called instrument errors. The expression for σ^2^ can alternatively be written as [(σ^2^_dark_ + σ^2^_read_) + (σ^2^_bg_ + *I*)] + (*gI*)^2^, where *I* is the net intensity. Scaling programs such as *SADABS* (Sheldrick, 1996 ▶) use the error model σ^2^_corr_ = *K*[σ^2^ + (*g*〈*I*〉)^2^] to obtain more reliable error estimates from internal standard deviations such that χ^2^ = 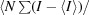 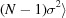 is close to 1.0. An incorrectly estimated gain value will affect the estimated standard deviations and *I*/σ of reflections, but scaling programs will more or less correct for this, notably *via* the χ^2^ analysis afforded by the usually routine data redundancies achieved these days with modern area-detector apparatus. Nevertheless, scale factors and rejections may depend on using the correct value of the gain.

While the intrinsic measurement errors on intensity are around 5% for protein crystals, the model refinement *R* factors on *F* are often in the range 20–25%. Vitkup *et al.* (2002 ▶) showed that the major contributions to the gap between *R* factors and the measurement errors are caused by the lack of a proper description of anisotropic protein motions that can often not be determined owing to the limited resolution of the data. Future developments in techniques and software, taking into account diffuse scattering, may improve on this situation provided that raw diffraction data are available for testing.

## 6. The usefulness of reintegrating data   

**Figure 2:**
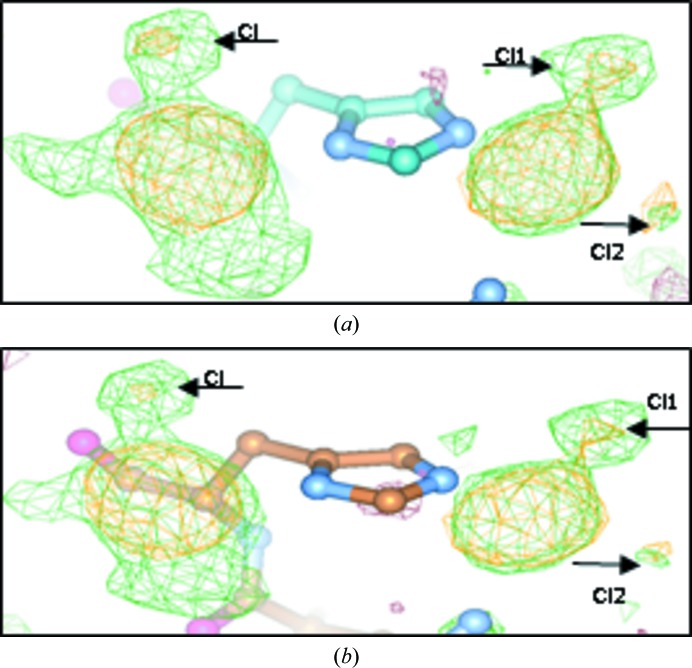
The two binding sites on the His15 residue of hen egg-white lysozyme (HEWL). The *F*_o_ − *F*_c_ OMIT electron-density maps are shown in green and the anomalous difference electron-density maps are shown in orange at a 3σ cutoff: 4dd7 (Tanley, Diederichs *et al.*, 2013*a* ▶) processed by (*a*) *XDS* (Kabsch, 2010 ▶) and (*b*) *EVAL* (Schreurs *et al.*, 2010 ▶).

We have recently written about another practical situation in which the raw X-ray diffraction data proved valuable and are now briefly reviewed. This is the example of Tanley, Diederichs *et al.* (2013*a* ▶) of locating a low-*Z* anomalous scatterer at fractional occupancy in a pharmaceutical chemistry crystallo­graphy study. Tanley, Diederichs *et al.* (2013*a* ▶) describe a partial chemical conversion of carboplatin to cisplatin under a high (10%) NaCl condition. This meant that they had the crystallographic challenge of observing partially occupied chlorine at a wavelength for X-ray diffraction data collection of 1.54 Å (*i.e.* with a quite small *f*′′ of 0.7 electrons for a fully occupied Cl). This study showed the importance of open archiving of the diffraction data images, which allowed a wider comparison of software results from them than the original study (for details, see Tanley, Diederichs *et al.*, 2013*a* ▶). We think that another way to resolve the partial chemical conversion of carboplatin to cisplatin is by co-crystallization in bromide conditions (Tanley *et al.*, 2014 ▶). Fig. 2 ▶ illustrates the technical challenge.

Low-*Z* elements are especially difficult for protein X-ray crystallography. The classic case is the challenge of trying to identify a sodium ion *versus* a bound water. These can be resolved by the putative sodium–ligand distances (∼2.2 Å) and the expected octahedral environment *versus* a hydrogen-bonding interaction for a bound water (∼2.8 Å), provided that adequate structural precision is available, which of course is not always the case. Besides software improvements, of course, experimental developments continue apace. Thus, the expansion into the use of longer X-ray wavelengths is also a real help in such cases; for a short summary, see Helliwell (2004 ▶). Examples of new instruments include the long-wavelength MX beamline I23 at Diamond Light Source led by Dr Armin Wagner; I23 will optimize the anomalous signals from sulfur in proteins or phosphorus in RNA/DNA crystals needed where labelling to introduce anomalous scatterers is not feasible or is too time-consuming. A review of the developments in long-wavelength resonant elastic X-ray scattering in the physical sciences is given by Fink *et al.* (2013 ▶).

## 7. Conclusions   

We made a local raw X-ray diffraction image data archive available at Utrecht University (http://rawdata.chem.uu.nl), which was subsequently mirrored at the TARDIS raw diffraction data archive in Australia. Some data sets were retrieved from http://rawdata.chem.uu.nl held at Utrecht University by K. Diederichs for reprocessing with *XDS*, which led to the analysis of anomalous densities in carboplatin-bound lysozyme crystals and also to a re-evaluation of the resolution cutoff in the diffraction data.

We demonstrate that often one needs prior knowledge, evidently of how to read the (binary) detector format, but also on the setup of goniometer geometries. This raises concerns with respect to long-term archiving of raw diffraction data. Care has to be taken that in the future unambiguous information is available, *i.e.* one cannot simply ‘deposit the raw data’ without such metadata details.

In general, we anticipate further progress by the IUCr Commissions in clarifying the metadata needs to accompany the raw diffraction, scattering and spectroscopic data that are relevant to them. Secondly, the proactive efforts of authors at the ‘grassroots’ level with their publications, as we have shown with ours, and the IUCr Executive at the ‘top-down’ level should help to contribute to making such raw data available in general and diffraction data images in particular. This trend is likely to be increasingly appropriate in the ‘open-access’ era, which extends not only to the written word but also to the data as the firm platform on which published science is, or should be, based. Increased raw data availability will be a natural extension to our crystallographic community leadership these last decades, along with the astronomers (ICSU, 2011 ▶), of ensuring in an organized way that processed experimental data and derived data are available with our publications.

## Supplementary Material

Supporting Information.. DOI: 10.1107/S1399004713029817/dz5309sup1.pdf

## Footnotes

1 Supporting information has been deposited in the IUCr electronic archive (Reference: DZ5309).
