# Supplementary material for: Experiences with making diffraction image data available: what metadata do we need to archive?
Source: Acta Crystallogr D Biol Crystallogr. 2014 Sep 30;70(Pt 10):2502–9. doi: 10.1107/S1399004713029817 (PMC4187998; doi:10.1107/S1399004713029817)
Supplement: Supplementary file 1 [file d-70-02502-sup1.pdf]

Acta Crystallographica Section D

Volume 70 (2014)

Supporting information for article:

Experiences with making diffraction image data available: What metadata do we need to archive?

Loes M. J. Kroon-Batenburg and John R. Helliwell

A compilation of the discussion that K. Diederichs (KD), L. Kroon-Batenburg (LKB) and A. Schreurs (AS) had with respect to the 4g4c data is given below:

KD: "I am using 1.54184 as the wavelength and 60 um as pixel size (both just guessed based on googling)". **LKB**: "Yes, that is correct. For the wavelength we use 1.541838 (or rather ImhAlpha1=1.54056 ImhAlpha2=1.54439 ImhRatio=1.99674). Pixel size is 60.00024 um."

KD: "the omega scans can be processed easily. I've been trying to process the phi scans but I can't index them. Are they measured with the same distance and delta-phi? I tried to compare headers of the 02 dataset with the 01 dataset but I don't have the right tools - I've been using strings, imosflm, labelit, adxv, xds-viewer but none of them gives me any useful information, nor does it even display these frames (xds does read them, but it does not use or print header information)! Is there any (preferably Linux) program that can read them, and tell me all the stuff one needs? I guess the rotation axis is tilted compared to an omega scan? By what amount?"

LKB: "There could be two reasons for your problems with this data set.
1) I suspect that you might have been too early in copying the 4g4c data from our web-site. Bruker software actually makes an error in writing the header, i.e. they do not adhere to their own description of the Bruker format. To correct for that, so that other software package apart from SAINT and EVAL can read the data, Toine wrote a Python script to convert the header information. He initially forgot to do that, so that you may have downloaded the wrong files.
2) The goniometer set-up of this equipment is rather weird. In the first phi-scan the rotation axis is tilted with respect to the primary beam, they make an angle of 140 degrees. In the second phi-scan the angle is 110 degrees. These are in fact very inefficient scans, with respect to the pass through the Ewald sphere, but also the beamstop is oriented in the equatorial plane, thus hiding the "best" reflections."

KD: "The header by itself would not prevent XDS from processing, since XDS does not read the header (for some detector types it does extract nx,ny and pixel size). I had not expected the type of geometry you describe. This means that the rotation axis is not perpendicular to the beam ... what XDS needs for processing are the components of the rotation axis w.r.t. the laboratory x, y and z axes. x and y are usually defined by the detector axes of the non-swung-out detector, and z is the beam direction. I believe there will be a lot of trial-and-error involved on my side in coming up with these 3 numbers. Unfortunately, I don't currently have the time for this. How shall we proceed? If mosflm can process these data then I can probably grab the required values from its logfile. Does EVAL print out that information?" **LKB**:" EVAL gives these numbers in our lab system: X= primary beam (from x-tal to source is positive), Z=Zenith (points upwards), Y= complements the right-handed system.

Direction of rotation axis of scan 2: -0.768688 0.277654 0.576218. Direction of rotation axis of scan 6: -0.345534 0.740661 0.576218."

KD (to the Manchester group): "One point though, having to do with the question of how to adequately document such data: I had problems processing the two phi-scans of 4g4c, because I could not deduce from the headers what the direction of the rotation axis was! Only with Loes' help I was able to process. Loes, do the headers really have that information, and how/where to find/deduce it? Or is additional information necessary, like the type of detector, the detector number, or some manual setting that is not recorded in the headers? For example, a phi scan for 4dd7 has a rotation axis just pointing in the opposite direction of the omega scan, but the header looks similar to me! What am I missing here? I tried processing the 4g4c phi scans with imosflm but that also failed. Maybe it is "just" a Bruker-related problem or maybe it is not important once you know how the data from "your" detector can be processed, but it's a nightmare if one wants to reproduce the data processing of datasets from unknown sources."

LKB: "I confirmed with Toine that the rotation axis could be derived from the header information of the Bruker FIXED CHI images. All goniometer geometries are given in Eulererian angles. "

KD: "Good to hear - that's a relief. Toine, could you please elaborate how to convert the angles given in the header to a rotation axis?"

AS: "This is how I handle the Bruker goniostat setup.

Our laboratory system: Origin at crystal; x-axis points to xray-source= (1,0,0); z-axis points to zenith = (0,0,1); y-axis makes it righthanded = (0,1,0). A positive rotation of 90 degrees around the z-axis rotates the x-axis to the y-axis. A standard euler setup: if all goniostat angles are zero: omega is along z-axis (pointing to zenith); chi is along x-axis (pointing to xray-source); phi is along z-axis (pointing to zenith). Unfortunately, Bruker is using a different scheme. First, the rotation direction of omega differs from the directions of chi and phi.

axis	rotdir	name
omega	1	RotDirOmega
chi	-1	RotDirChi
phi	-1	RotDirPhi

More unfortunately, if omega is zero, the chi-axis does not point to the xray-source but lies along the primary beam. In my goniostat routines, I use the original eulerian setup: if all goniostat angles are zero: omega is along z-axis (0,0,1); chi is along x-axis (1,0,0); phi is along z-axis (0,0,1)

To compensate for the Bruker setup, I add 180 degrees to omega. So if the header gives omega=0, I

use omega=180 and the chi axis at this position becomes (1,0,0)"

KD could successfully index and integrate the phi-scans with this information.
